# Current News and Opinion

**Published:** 1887-05

**Authors:** 


### Current News and Opinion.

### THE AMERICAN DENTAL ASSOCIATION.

*Editor Independent Practitioner:*

As a member of the American Dental Association, and one who first proposed Asheville, N. C , as the next place of meeting, I feel that the time has come for me to say something in regard to Dr. Field's letter in the last number of this journal. The spirit that prompted me to name Asheville was a desire for the good of the whole profession and the American Dental Association. The place was not an unwise selection. Its accessibility, its natural advantages, delightful climate, beautiful scenery, and its bracing atmosphere make it one of the most desirable places in the whole country, surpassed by none and equaled by few places in the South. The assertion that it has an uncomfortable climate and lack of hotel accommodations is quite a mistake. There are several good hotels and numbers of private boarding houses, which entertain summer visitors at reasonable rates. I have spent a month at a time there during July and August, and have a practical knowledge of the matter. The Southern Dental Association met with the North Carolina Dental Society there in 1881, and there was about as large an attendance as I have seen at the American for the past three or four years, and we had no trouble about entertainment. The American Medical Association and other bodies, both lay and professional, have met there and been well cared for. The place is quite flourishing, and is improving rapidly, with a resident population of seven or eight thousand.

There is one passage in Dr. Field's letter which deserves our consideration, and I confess has set me to thinking. It is this: "Will either the American Dental Association or the Dental Section of the Congress be apt to prove a complete success if both are held in the same year, and with so short a time intervening between the meeting?" I must admit, from the present lights before me, that I do not think they will. I have been one of those, from the first, who have never wanted or believed in this Dental Section, and I refused a place in its organization. Good and true men have, however, thought differently, and have exerted their energies to make a success of it. I find that eleven out of the twenty-six past presidents of the American Dental Association are now officers and members in this section, and there are thirty-six or more active members of the section and council who are now active members of good standing in the American Dental Association. We owe something to these men, and should pay deference to their wishes. I believe that a meeting of the Association this year would be detrimental, and I shall vote to postpone it one year, so that the members will not feel that there is a conflict of duty about attending either the one or the other. I know there are several propositions to change the time and place of meeting, but after carefully considering the matter, I believe that we shall not have a full or profitable meeting this year, wherever we may meet.

The Southern Dental Association cannot well meet with the American Dental Association, as it has already fixed upon its time and place, and they have shaped matters so that they may go directly from this meeting to the Congress. This would prevent their meeting with us at Asheville. If we should hold the meeting, and there should be a poor representation from the South, it would be charged that southern men were unwilling to meet with their northern brethren. If, however, the meeting be held at Asheville in 1888, there will, in my opinion, be one of the best and largest gatherings of dentists which America has ever seen. G. W. McElhaney.

Columbus, Ga., April 12, 1887.

---

### MASSACHUSETTS DENTAL LAW.

The following bill has been passed by the Legislature of the State of Massachusetts, and received the sanction of the Governor April 1st. Since the approval of the bill the Governor has appointed the following as members of the Board of Registration :

| | | | |
|---|---|---|---|
| For three years, | L D. Shepard, D. M. D., | Boston |
| " two " | J. S. Hurlburt, D. D. S., | Springfield. |
| " " " | E. V. McLeod, D. D. S., | New Bedford. |
| " one year | G. E. Mitchell, | Haverhill. |
| " " " | J. T. Dowsley, | Boston |

### An Act to Establish a Board of Registration in Dentistry.

*Be it enacted by the Senate and House of Representatives in General Court assembled, and by the authority of the same, as follows:*

Section 1. The Governor of the Commonwealth, with the advice and consent of the council, shall appoint, after the passage of this act, five skilled dentists of good repute, residing and doing business within the Commonwealth, who shall constitute a Board of Registration in dentistry; but no person shall be eligible to serve on said board, unless he or she shall have been regularly graduated from some reputable medical or dental college duly authorized to grant degrees in dentistry, or shall have been engaged in the practice of dentistry for a period of not less than ten years previous to his appointment : *provided, however*, that no person shall be eligible to serve on said board who is in any way pecuniarily connected with any dental college or dental department of any college or university. The term for which the members of said board shall hold their office shall be three years, except that two of the members of the board, first to be appointed under this act, shall hold their office for the term of one year, two for the term of two years and one for the term of three years respectively, and until their successors shall be duly appointed and qualified. In case of a vacancy occurring in said board such vacancy shall be filled by the Governor in conformity with this section. Any member of said board may be removed from office for cause, by the Governor, with the advice and consent of the council.

Sec. 2. Said board shall choose one of its members president, and one secretary thereof, and it shall meet at least twice in each year. Four of said board shall constitute a quorum, and the proceedings thereof shall, at all reasonable times, be open to public inspection.

SEC. 3. Within six months from the time this act takes effect, it shall be the duty of every person who is at that time engaged in the practice of dentistry in this State, to cause his or her name, residence and place of business to be registered with said board, who shall keep a book for that purpose. The statements of every such person shall be verified under oath before a notary public or justice of the peace in such manner as may be prescribed by the board. Every person engaged in the practice of dentistry within this Commonwealth at the time of the passage of this act, and who shall so register with said board as a practitioner of dentistry, shall receive a certificate to that effect, and may continue to practice without incurring any of the liabilities or penalties provided in this act.

SEC. 4. All persons not provided for in section three may appear before said board at any of its regular meetings and be examined, either orally or by written examination at the option of the several applicants, with reference to their knowledge and skill in dentistry and dental surgery; and if the examination of any such person or persons shall prove satisfactory to said board, the board shall issue to such persons as it finds to possess the requisite qualifications, a certificate to that effect, in accordance with the provisions of this act. All certificates issued by said board shall be signed by its officers; and such certificates shall be *prima facie* evidence of the right of the holder to practice dentistry in Massachusetts.

SEC. 5. Any person who shall violate any of the provisions of this act shall be deemed guilty of a misdemeanor, and, upon conviction, may be fined not less than fifty nor more than one hundred dollars, or confined three months in the county jail, for each and every offence.

SEC. 6. The said board shall charge each person receiving a certificate the sum of fifty cents, and each person appearing before them for examination for a certificate of qualification a fee of ten dollars, which fee shall in no case be returned. Any person failing to pass a satisfactory examination shall be entitled to be re-examined at any future meeting of the board, free of charge, but no applicant shall be examined oftener than twice in one year. Said board shall make an annual report of its proceedings to the Governor, by the thirty-first day of December in each year. All fees received by the board under this act shall be paid by the secretary of the board into the treasury of the Commonwealth once in each month.

SEC. 7. The compensation, and all necessary expenses of the board, shall be paid from the treasury of the Commonwealth. The compensation of the board shall be five dollars each for every day actually spent in the discharge of their duties, and three cents per mile each way for necessary traveling expenses in attending the meetings of the board, but in no case shall any more be paid than was actually expended. Such compensation and expenses shall be approved by the board and sent to the auditor of the Commonwealth, who shall certify to the Governor and council the amounts due as in case of all other bills and accounts approved by him under the provisions of law : *provided*, that the amount so paid shall not exceed the amount received by the treasurer and receiver-general of the Commonwealth from the board in fees as herein specified, and so much of said receipts as may be necessary is hereby appropriated for the compensation and expenses as aforesaid.

SEC. 8. Any person who shall falsely claim or pretend to have or hold a certificate of license, granted by any board organized under, and pursuant to, the provisions of this act, or who shall falsely, and with intent to deceive the public, claim or pretend to be a graduate from any incorporated dental college, or who shall practice dentistry without obtaining a certificate as provided in this act, shall be deemed guilty of a misdemeanor, and shall be liable to the same penalty as provided in section six.

SEC. 9. Nothing in this act shall apply to any practicing physician who is a graduate from the medical department of any incorporated college.

SEC. 10. This act shall take effect upon its passage.

---

### COMPLIMENTARY DINNER.

A dinner commemorative of the fiftieth year of dental practice of Dr. John B. Rich, of New York, was given at Mazetti's on the evening of February 26th, twenty-five dentists being present.

Dr. Rich was the first speaker, and recited his dental experience of half a century, calling attention to the fact that he was the connecting link between the past and present of dentistry in this country. He was personally acquainted with Dr. Greenwood, the first American dentist, and was present when the first dental college, and the first dental society, and the first dental journal were conceived, and in this connection related the following interesting fact: When Dr. C. A. Harris came to New York from Baltimore to endeavor to induce some medical college to open a dental department, having already failed in the South, he received no encouragement here. At one of the meetings, Dr. Solyman Brown suggested: "Why not establish an independent dental college?" Dr. Eleazar Parmly answered, "Yes; why not?" and Jehial Parmly, in his gruff and emphatic manner, added, "Certainly; why not?" Dr. Rich further stated that at a subsequent meeting Dr. Solyman Brown said: "And now let us have an independent dental journal!" and the journal and society immediately followed. Thus was born the first society, the first college and the first journal, and these marked the beginning and commenced the history of modern dentistry.

Drs. Dwinelle, Kingsley, Northrop, Jarvie and Perry were called upon, and each congratulated the venerable guest of the evening upon reaching this unusual anniversary, and upon the wonderful professional changes which he had been permitted to see, and to which he had contributed in so great a degree. Later in the evening some of the younger members of the profession were called upon, and Drs. Rhein, J. G. Palmer and Parmly Brown were not lacking in expressions of congratulation and good wishes.

It was a memorable occasion. Good cheer held smiling sway, and wine and wit sparkled spontaneously. With nothing to mar the harmony of the occasion, the guests at a late hour separated, wishing the venerable doctor many more annual anniversaries of his entrance upon dentistry. E. P. B.

### DENTAL SOCIETY OF THE STATE OF NEW YORK.

The nineteenth annual meeting will be held in the Common Council Chamber, Albany, Wednesday and Thursday, May 11 and 12, 1887.

The Society will be called to order at 10 A. M., Wednesday. Members, or others, who desire to present papers, the titles of which do not appear in the programme, should so inform the Chairman of the Business Committee, before the meeting. Persons intending to compete for the "Whitney Memorial Prize" must have their essays with the Chairman of the Committee on Prize Essays, Dr. M. D. Jewell, Richfield Springs, at least ten days before the meeting The envelope containing the essay should be marked "For the WHITNEY MEMORIAL PRIZE." The name of the writer may accompany the essay, *but in another and sealed envelope.* Conditions—The essay shall be worthy and the prize available.

The Secretaries of the several District Societies are requested to forward their reports to the Secretary of the State Society immediately. It is desired that the reports be made as complete as possible. For models, see transactions for '86. Persons who wish to exhibit appliances, materials, etc., should correspond with Dr. C. E Baxter, Albany.

The Board of Censors will meet at the Delevan House, Tuesday morning, May 10th, at 10 o'clock, for the examination of candidates for the degree of "Master of Dental Surgery" (M. D. S.). No examination will be held during the meeting of the Society. Information as to the requirements of the Board may be had by addressing the Secretary, Dr. Frank French, Rochester.

---

### EIGHTH DISTRICT DENTAL SOCIETY.

The nineteenth annual meeting of the above society was held in Buffalo, April 19th and 20th. The sessions were more than usually interesting, and profitable papers were read by Drs F. W. Low, "The Dividing Line"; H. A. Birdsall, "Dentistry versus Medicine"; W. C. Hayes, "Neuralgia"; W. A. Barrows, "Restoring Discolored Teeth"; M. H. Dailey, "Failures"; B. Rathbun, "Soft Gold in Filling Teeth." Some of them were of more than usual ability and interest.

Drs. C. J. Ellis, of Buffalo, and M. H. Lennox, now of Minneapolis, were expelled for unprofessional conduct.

Dr. C. C. Carroll, of Meadville, Penn., gave a clinic in the use of Aluminum as a base for artificial teeth, which attracted much attention and elicited favorable comment.

The officers elected to serve for the ensuing year are as follows:

*President*—J. Granger, Mayville.

*Vice-President*—F. E. Howard, Buffalo.

*Recording Secretary*—S A. Freeman, Buffalo.

*Corresponding Secretary*—B. Rathbun, Dunkirk.

*Treasurer*—C. W. Stainton, Buffalo.

*Librarian*—M. B. Straight, Buffalo.

*Censor*—L. W. Bristol, Lockport.

*Delegates to the State Society*—M. H. Dailey, G. B. Scott and J. Granger.

### ILLINOIS STATE DENTAL SOCIETY.

The Executive Committee of the Illinois State Dental Society announces the following list of reports and essays for the next annual meeting, which will be held at Jacksonville, the second Tuesday in May:

1.—Report of the Committee on Dental Science and Literature: Dr. C. R. E. Koch, of Chicago (Chairman); Dr. M. L. Hanaford, of Rockford; Dr. Louis Ottofy, of Chicago.

2.—Report of the Committee on Dental Art and Inventions: Dr. J. A. Swasey, of Chicago (Chairman); Dr. W. T. Magill, of Rock Island; Dr. J. Frank Marriner, of Ottawa.

3.—Essay—Dr. Norman J. Roberts, of Waukegan—"Regulating Appliances."

4.—Essay—Dr. Homer Judd, of Alton—"Retention of Pulpless Teeth in the Jaws."

5.—Essay—Dr. A. W. Harlan, of Chicago—"Practical Therapeutics, with Notes on the Application of Special Drugs."

6.—Essay—Dr. L. C. Ingersoll, of Keokuk, Iowa—"Medicinal Stimulants."

7.—Essay—Dr. Truman W. Brophy, of Chicago—"Diagnosis of Oral Tumors."

8.—Essay—Dr. W. N. Morrison, of St. Louis, Mo.—"Operative Dentistry as applied to Deciduous Teeth."

9.—Essay—Dr. L. L. Davis, of Chicago—"The Microscope and its Uses in Progressive Dentistry."

The culture of micro-organisms will be continued by Drs. Black and Moody, and an essay is expected from one of them, embracing the result of their investigations.

The clinics, under the supervision of Dr. C. F. Mattison, are expected to be unusually interesting.

In order that the report of the Committee on Mechanical Art and Inventions may be a valuable feature to our meeting, all of the members who have anything new, coming under that head, are urged to send or report it to the committee.

E. J. Greene,
W. H. Taggart,
P. J. Kester,
Executive Committee.

---

### KENTUCKY STATE DENTAL ASSOCIATION.

The seventeenth annual meeting will be held at Louisville, Ky., beginning Tuesday, June 7th, and continuing three days.

Members of the dental profession at large are cordially invited to attend, and unite with us in making the meeting one of great profit.

Reduced rates have been arranged for at the following hotels: Louisville Hotel, $2.50 per day; Fifth Avenue, $1.50 for two in a room, or $1.75 for one in a room, per day; St. Cloud Hotel, $1.50 per day; Alexander, $2.00 per day.

For further information address the Secretary,

Chas. E. Dunn, D. D. S.,
No. 529 Second Street, Louisville, Ky.

### CHICAGO COLLEGE OF DENTAL SURGERY.

Fifth annual commencement at the Grand Opera House, Monday, March 28, 1887, at 2.30 P. M.

### GRADUATES.

Bacon, Dewitt Clinton........Illinois.
Bentley, Charles Edwin...Wisconsin.
Calkins, Charles D., M. D....Illinois.
Conn, Walter Scott..........Illinois.
Davis, Ernest Edward......Michigan.
Dodge, Frank Armstrong. New York.
Hart, Edmund Jerome....Wisconsin.
Henderson, Luther David..Wisconsin.
Liggett, John...............Illinois.
Morris, William Evans.......Illinois.
Norton, M. Eugene...........Illinois.
Pagin, James Richard.......Indiana.
Reed, John Henry........Wisconsin.
Seeglitz, Otto Eberhardt......Illinois.
Underwood, Chester James...Illinois.
Wadsworth, Henry Palmer...Illinois.
Wermuth, Frank Charles..Wisconsin.
Wilson, Harry H............Illinois.
Zinn, Frank H...........Wisconsin.
Ballard, Henry Cliff.......Minnesota.
Broadbent, Thomas A., B. S..Illinois.
Coltrin, Charles Wilkins......Illinois.
Damon, William Henry......Illinois.
Deming, Charles Perry ...Wisconsin.
Goodearle, Joseph Henry..Wisconsin.
Haskins, George William.....Illinois.
Keefe, James Eucherius......Illinois.
Mawhinney, Elgion......Dakota Ter.
Nelson, Arthur.............Missouri.
O'Brien, Henry .............Illinois.
Pitt, Harry Norris...........Illinois.
Rosenkranz, Charles C.......Illinois.
Stover, Frank Garner........Illinois.
Wade, Harry Elmer.........Illinois.
Waschkuhn, Julius Albert... Illinois.
West, George Nelson.........Illinois.
Witt, William...............Illinois.

---

### CHICAGO DENTAL COLLEGE.

The fourth annual meeting and banquet of the Alumni Association of the Chicago College of Dental Surgery was held at the Leland Hotel, Saturday, March 26, '87, about sixty-five members being present.

The officers elected for the ensuing year were as follows:

*President*—Dr. E. E. Cady.
*First Vice-President*—Dr. J. A. Dunn.
*Second Vice-President*—Dr. J. G. Emery.
*Secretary*—Dr. James Stewart.
*Treasurer*—Dr. Edmund Noyes.

---

### FIRST DISTRICT SOCIETY OF NEW YORK.

The annual election of this society was held on Tuesday evening, April 5th, and the following officers chosen:

*President*—Dr. W. W. Walker.
*Vice-President*—Dr. J. F. P. Hodson.
*Secretary*—Dr. B. C. Nash.
*Treasurer*—Dr. C. W. Miller.
*Executive Committee*—Drs. A. L. Northrop, C. E. Francis and W. Carr.
*Censors*—Drs. E. A. Bogue, W. A. Bronson, C. A. Woodward, Frank Abbott and A. L. Northrop.

### CHICAGO DENTAL SOCIETY.

At the annual meeting of the Chicago Dental Society, held Tuesday evening, April 5th, the following officers were elected for the ensuing year:

*President*—Dr. J. G. Reid.

*First Vice-President*—Dr. J. A. Swasey.

*Second Vice-President*—Dr. G. H. Bentley.

*Recording Secretary*—Dr. C. N. Johnson.

*Corresponding Secretary*—Dr. W. B. Ames.

*Treasurer*—Dr. E. D. Swain.

*Librarian*—Dr. A. W. Harlan.

*Board of Directors*—Drs. G. H. Cushing, E. Noyes and J. A. Swasey.

*Board of Censors*—Drs. B. L. Rhein, J. W. Wassall and L. L. Davis.

W. B. Ames, Cor. Sec'y.

### THE AMERICAN ACADEMY OF DENTAL SURGERY OF NEW JERSEY.

The following is the list of officers elected at the annual meeting for the current year:

*President*—W. W. Walker, New York.

*Vice-President*—Fred. A. Levy, Orange.

*Secretary*—Chas. A. Meeker, Newark.

*Treasurer*—A R. Eaton, Elizabeth.

*Curator*—James G. Palmer, New Brunswick.

*Board of Examiners*—Fred. C. Barlow, Jersey City; Worthington Pinney, Newark; C. A. Timme, Hoboken; William P. Richards, Orange; G. Carleton Brown, Elizabeth.

*Counsel*—Herbert Boggs, 800 Broad Street, Newark, N. J.

### NEW YORK ODONTOLOGICAL SOCIETY.

The annual dinner of this excellent and well known society was held on Tuesday afternoon, April 12th, at Hotel Brunswick, on Fifth Avenue. It was a very pleasant gathering, and a number of distinguished guests from other States were present.

The dinner over, and after many friendly greetings, the party started for the Academy of Medicine, where a feast of another character was partaken and apparently enjoyed by those who had the good fortune to listen to the reading of Dr. Davenport's excellent paper, and the discussions that followed.

### MASSACHUSETTS DENTAL SOCIETY.

The semi-annual meeting will be held at the Hawthorne Rooms, No. 2 Park Street, Boston, Thursday and Friday, June 9 and 10, 1887.

The committee expect this to be one of the most valuable and instructive meetings ever held.

Papers and clinics of particular interest are already assured.

Per Order Ex. Com.,

Geo. F. Eames, Sec'y.

### HYDRONAPHTHOL.

This, the latest antiseptic and disinfectant obtained from the phenol series of coal tar products, is rapidly forging its way to the front in popular estimation. Surgeons and gynæcologists everywhere, both in hospital and private practice, accord it the highest praise for all purposes of an antiseptic and disinfectant. Its freedom from odor, poisonous properties or corrosiveness, gives it preference over carbolic acid and all antiseptics heretofore employed. A book containing full description of Hydronaphthol will be mailed free to physicians applying by postal to Seabury & Johnson, Manufacturing Chemists, 21 Platt St , New York.

---

### KANSAS STATE DENTAL ASSOCIATION.

The sixteenth annual meeting will be held at Topeka, Kansas, commencing Tuesday, April 26, 1887, and will continue three days. It is expected that this will be one of the best meetings of the society ever held, and a cordial invitation is extended to all to be present and assist in making it such.

C. B. Gunn, Secretary.

---

### MISSOURI STATE DENTAL ASSOCIATION.

The twenty-third annual meeting of the Missouri State Dental Association will be held at Kansas City, Mo., the last Tuesday in June, continuing four days, June 21st and 24th inclusive. John G Harper, D. D. S , Rec. Sec'y.

William Conrad, D. D. S., President

---

When Dr. McKellops does anything, it is apt to be well done. His latest evolution is a "Dental Blotter" that is marvelously convenient. In the first place, on each leaf is a diagram of all the permanent teeth, and the first of its kind, so far as our knowledge extends, which clearly exhibits every surface of every tooth, with all the roots in perfect contour, so that an operation may be distinctly marked in its exact location. On the same leaf is a proper form for recording the name, residence, etc., of the patient, and below this a blank ruled for accounts. Then the whole is perforated, so that it may easily be torn out and sent as a bill, the patient thus having a perfect record of the case. It is thus a bill-book, a dental record and blotter, all in one.

During a recent visit to the editor, Dr. McKellops explained the method of using it, and it would really seem to be almost indispensable to a systematic method of keeping accounts. He purposes, as soon as leisure can be found, to prepare a "Dental Register" upon the same general principle.

---

The Dentists of Buffalo met at the office of Dr. Southwick, on the evening of April 12th, to take into consideration the propriety of establishing a dental college in Buffalo. After a full and free interchange of opinion a ballot was taken, which resulted in but one vote in favor of establishing a college. So it may be understood that the disinterested dentists of Buffalo do not believe that a college is indispensable, either for their prosperity or happiness, or for the good of dentistry.

The Seventh District Dental Society held its annual meeting in Rochester, on Tuesday and Wednesday, April 26th and 27th. The following were elected officers for the ensuing year:

*President*—F. A. Greene, Geneva.

*Vice-President*—F. D. Browne, Mt. Morris.

*Recording Secretary*—C. T. Howard, Rochester.

*Corresponding Secretary*—W. A. White, Phelps.

*Treasurer*—J. Requa, Rochester.

The meeting was well attended, and the papers and discussions were more than usually profitable.

---

Scribner's Magazine ! The words have a refreshing sound. Many thousands of the readers of the old *Scribner's*, as edited by Dr. Holland, were disappointed when that journal was changed to *The Century*, and have never ceased to regret their old favorite. To such, the knowledge that *Scribner's* has been revived, will come with welcome sound. The new series bears many of the features which made the old *Scribner's* such a favorite. We miss the genial spirit and graceful pen of the author of "Bitter Sweet," but his place is well filled by others. Welcome ! thrice welcome ! then, to the new *Scribner's !*

---

Dr. J. Austin Dunn, of Chicago, has devised the very best of the many medicinal syringes which have been presented to dentists. It is so simple and yet so effectual that the only wonder is that it was not sooner conceived. The long, delicate, yet strong and flexible point may be thrust up nerve canals, or be inserted deeply into an alveolar abscess, and the force with which remedies may be thrown is quite surprising. It is essential to a perfect practice. It will be found illustrated in the advertising pages.

---

The Open Court is the name of a fortnightly paper published in Chicago, which is the index of free thought in religious and scientific matters. It is not the organ of freethinkers in the sense of free license to revile everything good, but of advanced and rational religious thought, in contradistinction to religious bigotry, intolerance and pharisaism—of a religion that is not in direct conflict with the truths of science. It is an excellent journal for a thinking man.

---

Dr. E. Parmly Brown is a very ingenious man, and has invented many appliances which are essential to many dentists. It would be a difficult matter to enumerate them all, but if the reader will turn to the advertising pages of this number, he will find the claims of some of the more important ones set forth. The editor of this journal has personally tested the most of them, and can vouch for their practicability and usefulness.

---

Among the successful works of fiction of the present season are Dr. S. Weir Mitchell's "Roland Blake" and Dr. William A. Hammond's "Tales of Eccentric Life."

---

Dr. Wilhelm Herbst, of Germany, has been honored with the diploma of the Cincinnati Dental College.

